# Are Engaged Workaholics Protected against Job-Related Negative Affect and Anxiety before Sleep? A Study of the Moderating Role of Gender

**DOI:** 10.3390/ijerph15091996

**Published:** 2018-09-13

**Authors:** Paola Spagnoli, Cristian Balducci, Liliya Scafuri Kovalchuk, Francesco Maiorano, Carmela Buono

**Affiliations:** 1 Department of Psychology, University of Campania Luigi Vanvitelli, CE 81100 Caserta, Italy; liliya.scafurikovalchuk@unicampania.it (L.S.K.); francesco.maiorano91@gmail.com (F.M.); carmela.buono@studenti.unicampania.it (C.B.); 2 Department of Psychology, Alma Mater Studiorum University of Bologna, BO 40126 Bologna, Italy; cristian.balducci3@unibo.it

**Keywords:** workaholism, work engagement, job-related negative affect, sleep disorders, gender

## Abstract

Although the interplay between workaholism and work engagement could explain several open questions regarding the Heavy Work Investment (HWI) phenomenon, few studies have addressed this issue. Thus, with the purpose of filling this literature gap, the present study aimed at examining a model where job-related negative affect mediates the relationship between the interplay of workaholism and work engagement, and anxiety before sleep. Since gender could have a role in the way the interplay would impact on the theorized model, we also hypothesized a moderated role of gender on the specific connection concerning the interplay between workaholism and work engagement, in relation to job-related negative affect. Conditional process analysis was conducted on a sample of 146 participants, balanced for gender. Results supported the mediating model and indicated the presence of a moderated role of gender, such that engaged workaholic women reported significantly less job-related negative affect than disengaged workaholic women. On the contrary, the interplay between workaholism and work engagement did not seem significant for men. Results are discussed in light of the limitations and future directions of the research in this field, as well as the ensuing practical implications.

## 1. Introduction

Several recent transformations in the working processes (e.g., strenuous competition, accelerated change and instability, intensified workload and requests for flexibility and continuous learning), coupled with the increasing use of information and communication technology (ICT) allowing people to work anytime and anywhere, have dramatically increased the risk for overwork, and for extreme, unsustainable and unhealthy forms of work investment, such as workaholism [1,2]. Workaholism, firstly defined by Oates [3] as an irresistible or uncontrollable need to work incessantly, is different from work engagement, which is a positive and healthy form of heavy work investment, and is correlated to high health impairment, such as job strain and sleep disorders [4,5,6,7,8,9]. Work-engaged employees work hard, are very involved in their work, and are happily engrossed in it, thus being similar to workaholics. However, they do not work hard because they cannot disengage from work, but because they enjoy their work [10]—that is, they lack the strong compulsive and maniac tendency to work hard that characterizes workaholics. Organizations need engaged individuals, not workaholic individuals. Unfortunately, the difference between the two phenomena is still poorly understood and there is concern that, with their way of operating, modern organizations may nurture workaholism, rather than work engagement.

Recent theoretical and empirical contributions on heavy work investment (HWI) have outlined a possible intriguing interplay between the negative form of HWI—workaholism—and the positive one—work engagement—in configuring different workaholic types, such as disengaged workaholics vs. engaged workaholics [6,11]. HWI was first conceived by Snir and Harpaz [12], who claimed that time and effort investments in work constitute the two core dimensions of HWI in general. The HWI concept incorporates these two core dimensions while eschewing a priori positive or negative associations to the outcomes. Thus, workaholism and work engagement can be seen as the two sides of the same coin. Although both engaged workaholics and disengaged workaholics share similar patterns related to the work–family outcomes, since they both spend an exaggerated span of time working at the expense of their extra-working life, they differ in the way they experience negative and positive emotions and other negative outcomes, especially those related to health and wellbeing, such as sleep disorders, given that engaged workaholics seem to be protected from experiencing any extreme unhealthy conditions. Although the interplay between workaholism and work engagement could explain several aspects connected to the comprehension of the HWI phenomenon, few studies have addressed the issue [11].

Moreover, although several studies reported sleep disorders as a possible negative outcome of workaholism [8,9,12,13,14], the process explaining this relationship remains unrevealed. Actually, work-related sleep disorders are very common and may have significant short- and long-term effects on health and safety [15,16].

**Figure 1:**
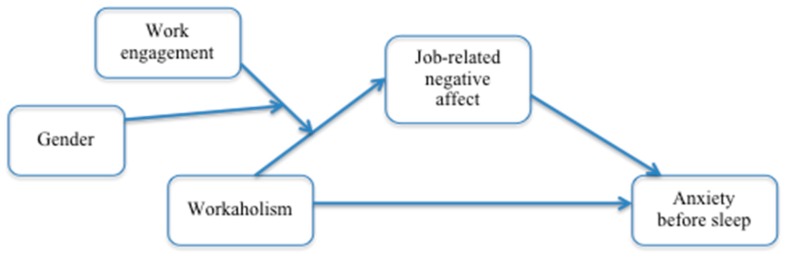
Mediation effect of job-related negative affect in the relationship between workaholism and anxiety before sleep including interaction between workaholism and work engagement on job-related negative affect, moderated by gender.

In the current study, we hypothesized a crucial effect of job-related negative affect in triggering this process. Thus, to contribute to this literature, the aim of present study was twofold: (1) we examined a model where job-related negative affect might mediate the relationship between workaholism and anxiety before sleep; and (2) we examined the influence of the interplay between workaholism and work engagement, moderated by gender, on the experience of job-related negative affect. Moreover, since workload is reported as one of the major correlates of workaholism, and it is also associated with negative affect [17] we included it in the study as a control variable. Theoretical and empirical support for designing the current study represented in Figure 1 is better elucidated in the following sections.

### 1.1. The Relationship between Workaholism and Sleep Disorders

One of the health consequences of being workaholic is a poor quality of sleep [8,9]. Several studies have investigated the relationship between being workaholic and sleep quality, although the methods in which the sleep quality was conceptualized and measured were different [8,9,18,19]. For instance, some studies [13,14] focused their attention on investigating the relationship between workaholism and insomnia, a disorder that affects a significant portion of the world population [20], reporting a significant and positive association between the two constructs. All these studies, despite the different conceptualization and measurement of sleep disorders, have reported a positive and significant association between these and being workaholic, emphasizing even more how negative the impact of workaholism can be on the quality of the worker’s sleep, which, if poor, determines, for example, a drop in cognitive functions, such as attention and the ability to reason or make judgments, a capacity clearly fundamental for any type of work [21]. In the current study, we focused on a particular sleep disorder, namely, anxiety before sleep.

Although several studies reported a significant association between workaholism and poor quality and quantity of sleep, research on the mechanism linking these variables is scant and fragmented. In the current study, we addressed this issue by positing a comprehensive model envisaging a possible mediating effect of job-related negative affect in the relationship between workaholism and anxiety before sleep. Because factors relating to anxiety and stress are one of the most important concomitants of sleep complaints in the general population [22], we believe that anxiety before sleep may also be one of the most significant negative outcomes of being workaholic.

### 1.2. The Mediating Role of Job-Related Negative Affect in the Relationship between Workaholism and Anxiety before Sleep

According to the HWI theoretical framework [12], workaholics not only devote a great amount of time to work, beyond what is reasonably expected of them, but they also expend high physical and mental energy on their work. The effort–recovery model [23] postulates that high energy expenditure at work is accompanied by sympathetic activation and load (or strain) reactions. Such condition has affective correlates, since emotions are fundamental components of the stress experience [24,25]. According to allostatic load theory (see, e.g., Gangster and Rosen [26]), strain manifests itself at the affective level by the experience of negative emotions such as anger, tension, anxiety and depression, which acts as primary mediators of the stress process. Thus, we assume that, by investing a very high effort at work, workaholics spend high levels of mental and physical energy and commonly report work-related load or strain reactions in the form of negative affective experiences. Recent studies indeed found a clear pattern of results regarding the relationship between workaholism and job-related negative affect [27,28].

Empirical research has shown that workaholism can be associated with increased trait negative affect [29], which, in turn, has significant and positive relationships with anxiety/insomnia, somatic health, emotional exhaustion, depressed mood, and social dysfunction [15,30,31]. In particular, anxiety about duties after working hours and/or during non-work days can be associated with difficulty falling asleep [32], because the persistent thinking about work, may result in sympathetic arousal and emotional distress. According to Kahn and colleagues [33], research has convincingly shown that stress and its associated negative emotions are related to sleep disturbances such as reduced sleep quantity and poor sleep quality as measured by self-reports, actigraphy, and polysomnography [34,35,36,37,38,39]. LeBlanc and colleagues [40], for example, have found that anxiety symptoms are a major risk factor for insomnia. Similarly, stress is the main etiological factor of primary insomnia according to the diagnostic classification of sleep disorders. Results regarding the association between negative affective states and sleep patterns are also confirmed by Vandekerckhove and colleagues [41] and Tang and Harvey [42], who showed that valence and arousal might both contribute to poor sleep.

Taking these findings together, the logical nexus between workaholism, job-related negative affect and poor sleep quality, seems evident. Thus, we see job-related negative affect as logical mediators of the relationship between workaholism and sleep disorders. Since previous studies have only examined direct relationships between workaholism, job-related negative affect and sleep disorders, and not these relationships together, the current study aimed to shed some light on this mechanism. Therefore, our first hypothesis was the following:

**Hypothesis** **1.** *Job-related negative affect will mediate the relationship between workaholism and anxiety before sleep.*

### 1.3. The Interaction between Workaholism and Work Engagement on Job-Related Negative Affect, Moderated by Gender

Excessive working may also reflect enjoyment and vitality, which is often labeled as work enthusiasm [43] or work engagement [44]. Work engagement is considered a persistent affective cognitive state of well-being that is rather pervasive [45] and not related to any specific objects or events [46]. The concepts of work engagement and workaholism share similarities as both are characterized by a heavy investment in work that is driven either by a strong sense of involvement and identification with the job (work engagement) or a strong inner urge to work very hard (workaholism) [47]. However, a general consensus exists that, whereas workaholism is primarily associated with negative outcomes, work engagement is usually linked with positive outcomes. For instance, engaged employees are more satisfied with their jobs and are more committed to the organization [7,48], show more personal initiative [49], exhibit more extra-role behavior and perform better [50,51], have a lower intention to leave the organization [52], and are less often absent [53] than disengaged employees. Further, engaged employees spend more time on socializing, hobbies, and volunteer work [54], experience high life satisfaction, and good mental and physical health [7,48,55].

Research on the relationship between workaholism and work engagement reported a relative independence of both concepts since they are usually uncorrelated [7,56]. According to some authors [6,11], this implies that four types of workers may be distinguished: (a) employees who are workaholic and disengaged (workaholic employees); (b) employees who are nonworkaholic and engaged (engaged employees); (c) employees who are both workaholic and engaged (engaged workaholics); and (d) employees who are nonworkaholic and disengaged (nonworkaholic/disengaged workers). The latter type of workers would refer to those who are satisfied with accomplishing the prescribed tasks without going beyond organizational requirements: they are satiated, rather than activated.

In particular, it can be assumed that disengaged workaholic employees frequently experience negative outcomes that are energy consuming and could impede the recovery process after working [6]. Conversely, engaged employees appear to be able to recover adequately from their work [49]. Interestingly, despite working harder than others, engaged workaholics experience less burnout than workaholic employees. Thus, apparently, work engagement could buffer against the adverse effects of workaholism, rendering engaged workaholics less vulnerable for developing burnout. Furthermore, Loscalzo and Giannini [11] believed that making a distinction between engaged and disengaged workaholics is very important since it can help prevent overpathologizing working itself, which is a common and often socially valued behavior. This issue was also recently raised by Billieux and colleagues [57], who criticized the current tendency to label virtually all human activities in terms of behavioral addictions and hence as clinical pathologies. Since work is an important daily activity, and given that the stigma in organizations that could be associated with a diagnosis of workaholism might be associated with negative effects, Loscalzo and Giannini [11] believed that it is important to make a clinical diagnosis of workaholism only when overworking is associated with low work engagement and high impairment, as usually required by the DSM criteria [57]. Thus, in accordance with Loscalzo and Giannini [11] and Van Beek and colleagues [6], in the current study, workaholism and work engagement were considered together and crossed conceptually to identify different kinds of workaholics. Two different kinds of workaholics were considered, engaged and disengaged workaholics, in relation to perception of job-related negative affect. Previous evidence indicates that workaholism is related to negative affect, whereas work engagement is associated with positive affect [29,58,59,60,61]. Accordingly, it is reasonable to suppose that disengaged workaholics and engaged workaholics will differ in the way they perceive job-related negative affect, so that the engaged component will protect/buffer against it. Thus, we put forward our second hypothesis:

**Hypothesis** **2.** *The interplay between work engagement and workaholism in relation to job-related negative affect will be significant.*

Furthermore, in the present study, we expected to find evidence of a gender effect on the interaction between workaholism and work engagement, relating to job-related negative affect. As gender is usually considered in the study of workaholism, we believe it could also play a role in the interaction between workaholism and work engagement relating to job-related negative affect. The theory of sex roles [62] and the cultural patterns associated with work and family devotion [63] suggest that for men it is socially acceptable (and expected) to be completely absorbed by work, while women are expected to be more involved in family commitments. This is obviously culturally dependent, since it is still a persistent phenomenon especially in the more traditional societies. Such beliefs and expectations about the different division of roles and responsibilities influence the behavioral patterns of men and women and can partially explain the gender differences obtained from the studies on workaholism. However, results on gender differences and workaholism are mixed. Some authors [64,65] identified in men a greater propensity to become workaholic. Some studies do not show any significant differences between genders in workaholism [4,66,67,68,69,70,71]. Other studies report higher scores for women in some workaholism components and higher scores for men in others [72,73]. Bueleus and colleagues [74] reported that women were more likely to experience workaholism. Thus, the available research did not draw a conclusive picture on the relationship between gender and workaholism. In the current study, a mediating role of job-related negative affect is supposed to link the interaction between workaholism and work engagement, and anxiety before sleep. Since it could be argued that women and men tend to express their emotions differently, gender differences could arise in terms of job-related negative affect. Some authors pointed out that women show a wide range of emotional expression [75,76,77], whereas men express more negative emotions such as anger and aggression [78,79]. Moreover, recent contributions by Banihani and colleagues [80] outlined that the construct of work engagement cannot be considered gender-neutral, as it is easier for men to demonstrate work engagement than for women. These authors concluded that the structure, culture, and ideologies of organizations place women at a disadvantage and make it harder for them to experience work engagement and that, to be engaged at work, women need to take on an extra workload. Thus, since work engagement is more difficult to achieve for women than for men, it is likely that, when women are engaged at work, the benefits would be higher than for men. In other words, because work engagement is more challenging for women than for men, it is possible that achieving it could be more rewarding for women, who consequently will experience more benefits in reducing the negative aspects of workaholism, such as job-related negative affect. Thus, we hypothesized that:

**Hypothesis** **3.** *Gender will modify the consequences of the interplay between workaholism and work engagement in terms of job-related negative affect, such that engaged workaholic women will experience less job-related negative affect than engaged workaholic men.*

## 2. Materials and Methods

### 2.1. Recruitment

The sample was selected based on the profession that the participants carried out, focusing on jobs at high risk of workaholism [81], and balancing it for gender.

### 2.2. Participants

A total of 146 Italian participants took part in the study. The sample is perfectly balanced for gender since it consisted of 73 women and 73 men. Their age ranged from 22 to 71 years (M = 45.65; St.D. = 11.614). Moreover, it is constituted by four main profession categories distributed as follows: teachers, 26%; doctors, 6.8%; managers, 48.6%; and freelancers, 18.6%. They were employed in both the private sector (56.2%) and public sector (43.8%). Educational level was distributed as follows: 4.1%, middle school; 39%, high school; and 54.8%, bachelor degree or more.

### 2.3. Questionnaire Administration

A self-report questionnaire was administered by hand delivery and returned through the snowball technique by involving graduated students on Work and Organizational Psychology courses who took part in the data collecting phase of the study as part of their master degree thesis assignment. Particular attention was dedicated to training the involved students. Students identified acquaintances in their social network with jobs at high risk of workaholism, and proposed them to take part in a study on work-related health and well-being issues.

### 2.4. Measures

#### 2.4.1. Workaholism

Workaholism was measured by using the 10-item version of the Dutch Work Addiction Scale (DUWAS) previously adapted and validated in Italy [82]. The DUWAS investigates the respondent’s feelings about his/her work, which reflect the two components of workaholism (i.e., working compulsively, WC, and working excessively, WE). Example items are the following: “*I feel that there*’*s something inside me that drives me to work hard*” (WC) and “*I stay busy and keep many irons in the fire*” (WE). Responses were given on a 4-point scale varying from 1 (“Never or almost never”) to 4 (“Almost always or always”). Similar to previous studies, the two workaholism components were strongly correlated (r = 0.57, *p* < 0.001). Thus, following the examples of others [6,27], in this study, we derived an overall workaholism score.

#### 2.4.2. Work Engagement

Work engagement was measured with the 9-item Utrecht Work Engagement Scale [83] adapted in Italy by Balducci and colleagues in 2010 [84]. Participants were asked to respond on a 5-point scale ranging from “never” to “every day” how frequently they experienced the feeling indicated by the nine items. Item example: “*In my work I feel strong and vigorous*”.

#### 2.4.3. Job-related Negative Affect

This was measured using the four negative affective states derived from the Job-related Affective Well-being Scale (JAWS) [85]. Participants were asked how much they had felt any of different negative (including high and low arousal) affective states during the last month, with responses given on a 5-point scale ranging from 1 (“not at all”) to 5 (“very much”). The negative affective states assessed were anger, anxiety, pessimism, and discouragement.

#### 2.4.4. Anxiety before Sleep

Anxiety before sleep was measured with four items adapted from the Sleep Disturbance Questionnaire [86]. Participants were asked to respond on a 5-point scale indicating the degree to which several anxiety indicators before sleep could disturb their sleep. An item example is: “*I feel very* ‘*agitated*’ *from not sleeping*”.

#### 2.4.5. Workload

Workload was measured by three items (e.g., “*I have to work very fast*”) from the Job Content Questionnaire [87]. Responses were given on a 5-point scale varying from 1 (“strongly disagree”) to 5 (“strongly agree”).

Since workaholism and work engagement scales were already adapted in Italian, a rigorous translation process was conducted only for the remaining scales following Brislin’s procedure of the back-translation [88].

### 2.5. Data Analysis

Zero-order correlations were used to examine associations between variables. Reliability analysis was used to assess the scales’ internal consistencies. Although the most commonly used statistic for evaluating reliability is Cronbach’s α coefficient, it bears some limitations. Therefore, a proper method using, for example, the parameter estimates in confirmatory factorial analysis using SEM is recommended [89,90]. Thus, reliability was tested using the method described by Rios and Wells [89]. Specifically, reliability resulted from the equation where the numerator represents true score variance and the denominator represents the total observed score variance and includes the true score variance, error variance and any non-zero correlated measurements errors [89].

In addition, Harman’s single-factor technique conducting a confirmatory factorial analysis with AMOS 20 (IBM, Armonk, NY, USA) was used to assess the risk for common method variance. This technique can be applied in a study, as the current one, that utilizes a single method to collect the data. A CFA could be used in this approach, wherein all the items are modeled as indicators of a single latent factor and common method variance is evidenced if that one factor were to best fit the data.

Fit indices assessed were: *X*^2^/*df*, CFI, and RMSEA. CFI assesses the extent to which the tested model is superior to an alternative model in reproducing the observed covariance matrix [91,92]. The CFI index varies from 0 to 1 and a cutoff criterion of CFI > 0.95 is needed to ensure that misspecified models are not accepted [93]. The RMSEA introduces a correction for lack of parsimony, given that, all other things being equal, more complex models are penalized. A cutoff value close to 0.06 [94], or a less stringent upper limit of 0.08 [95], seems to be the general consensus among the researchers in this area. The normed *X*^2^, or *X*^2^/*df*, is a further version of the traditional *X*^2^/*df*. The advantage of the *X*^2^/*df* is that it might be less sensitive to the sample size. The criterion for acceptance of the *X*^2^/*df* index varies across researchers, ranging from less than 2 [96] to 5 [97]. However, it has to be noted that the cutoff values of the fit indices cannot be interpreted as golden rules or even as a fixed value independent of the data given [98].

To test the hypotheses, two models were tested: Model 1 represents a simple mediation model where job-related negative affect mediates the relationship between workaholism and anxiety before sleep and Model 2 adds the interaction between workaholism and work engagement on job-related negative affect, moderated by gender, to Model 1. Workload, age, job sector, tenure, and gender were inserted in Model 1 as control variables. The same set of control variables, except for gender, which was examined in the model as moderator, was included in Model 2. The hypotheses concerning direct, mediated and moderated effects were tested through conditional process analysis based on OLS regression using bootstrapping technique [99], a nonparametric resampling procedure that does not assume normality and involves the extraction of several thousand subsamples (5000, in our case) from a dataset. Through bootstrapping, the distribution of effects is empirically approximated and used for calculating confidence intervals [100]. We calculated a “moderated moderated” mediation model to verify the existence of “moderated moderated” effects and conditional indirect effects depending on gender. Specifically, the model examined in the current study corresponds to the conceptual model number 11 of Hayes [99] templates.

### 2.6. Ethical Aspects

The procedure was in accordance with the standards of the national law of data treatment followed by the University of Campania (Italy). Since there was no medical treatment or other procedures that could cause psychological or social discomfort to participants, who were all adult healthy subjects anonymously involved, additional ethical approval was not required according to the Institution. The research was conducted in line with the Helsinki Declaration, as well as the data protection regulation of Italy (Legislative Decree No. 196/2003). Participation in the research was voluntary and not rewarded; data collection and analysis were anonymous. A cover letter attached to the questionnaire provided information about the study aims, guarantees about anonymity, voluntary participation and data treatment, and instructions for filling out the questionnaire. Agreeing to fill out the questionnaire, all study participants provided their informed consent.

## 3. Results

Table 1 shows the zero-order correlation among study variables and their reliability measured by reliability SEM analysis. Workaholism is positively and statistically related to job-related negative affect, anxiety before sleep and workload, whereas it is unrelated to work engagement. Reliability coefficients were all above 0.84 and thus satisfactory. Table 2 shows a series of one-way ANOVA that were conducted to check the gender effect on the study variables. Apparently, gender differences did not emerge, although, for workaholism and work engagement, the F values were close to statistical significance (*p* = 0.06). Before conducting the main analysis, Harman’s single-factor technique, adopting a confirmatory factorial analysis (CFA) was used to identify the risk for common method variance. Results of the CFA indicated a poor fit for a single factor including all the observed variables under study (*X*^2^/df = 5.996; CFI = 0.34; RMSEA = 0.19). We also conducted a CFA including all the variables under study as separate factors. Fit indices for this model were superior to those of the single factor model (*X*^2^/df = 1.744; CFI = 0.91; RMSEA = 0.07). Thus, according to our test, the hypothesized factors could be discriminated empirically, meaning that the CMB should not have contributed substantially to the emerged results. Table 3 reports the results for the conditional process analysis conducted on the two models.

Evidence of a significant partial mediating effect of job-related negative affect in the relationship between workaholism and anxiety before sleep was found (Indirect effect = 0.10 LLCI = 0.01 ULCI = 0.24) in Model 1. Workaholism and job-related negative effect explained 19% of variance of anxiety before sleep. Furthermore, none of the control variables had a significant effect in the tested model. Thus, Hypothesis 1, assuming that job-related negative affect could mediate the relationship between workaholism and anxiety before sleep, was supported.

Then, in Model 2, evidence of a significant interaction between workaholism and work engagement was found (B = −0.38 LLCI = −0.67 ULCI = −0.09) as well as a moderating effect of gender in the interaction between workaholism and work engagement on job-related negative affect (B = −0.80 LLCI = −1.40 ULCI = −0.22. As in Model 1, none of the control variables had a significant effect in the tested model.

Thus, Hypotheses 2 and 3 were supported.

**Figure 2:**
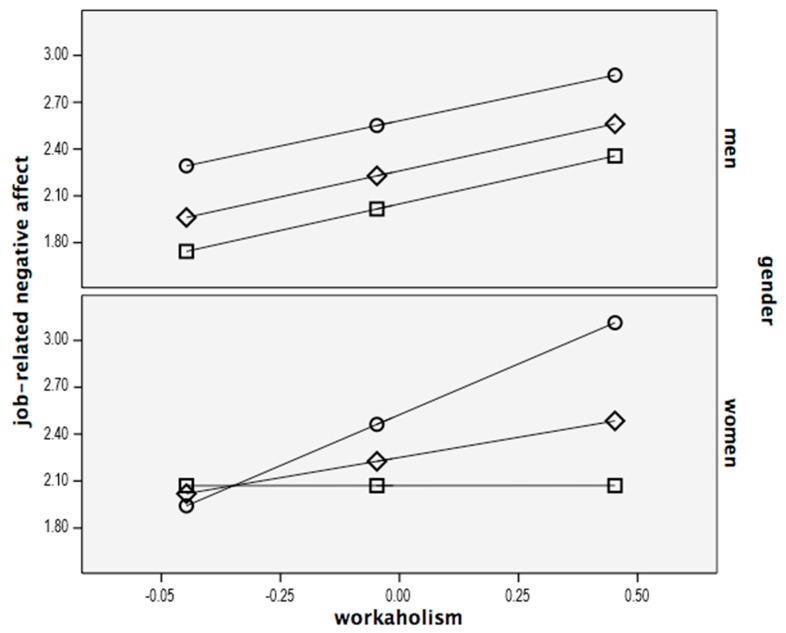
Plots of the interaction term between workaholism and work engagement on negative emotions moderated by gender. □ = high work engagement; ○ = low work engagement; ◊ = medium work engagement.

Figure 2 reports the plots regarding the interaction between workaholism and work engagement on job-related negative affect separately for women and men.

Following Hayes [99], the values of workaholism were observed at the 16th, 50th, and 84th percentile in work engagement. In the women’s plot, when work engagement is low and workaholism is high, job-related negative affect is significantly higher than when work engagement is high. In particular, simple slope analysis for women revealed that the relationship between workaholism and job-related negative affect was positive and significant (B = 1.30 *p* < 0.001) at low (i.e., 16th percentile) work engagement, while it was non-significant (B = 0.008, *ns*) at high (i.e., 84th percentile) work engagement. Thus, we can conclude that, among women, the relationship between workaholism and job-related negative affect is stronger when work engagement is low, compared to when work engagement is high. This suggests that work engagement acts as a protecting factor among women, as far as the relationship between workaholism and job-related negative affect is concerned. On the contrary, among men, the level of work engagement does not modify the relationship between workaholism and job related negative affect.

## 4. Discussion

The current study aimed at providing an empirical contribution on two main issues: the possible mediating role played by job-related negative affect in the relationship between workaholism and anxiety before sleep, and the effect of the interplay between workaholism and work engagement, likely moderated by gender, on job-related negative affect. These two issues were addressed in two mediating models: Model 1, where job-related negative affect played a mediating role in the relationship between workaholism and anxiety before sleep; and Model 2, where job related negative effect mediated the interplay workaholism/work engagement, moderated by gender, and anxiety before sleep.

As regards the study of the relationship between workaholism, job-related negative affect and anxiety before sleep (Model 1), we provided evidence of a possible mechanism through which workaholism could impact on one of its outcomes. Research has so far reported just a direct relationship between workaholism and sleep disorders, and the process linking these variables remains unrevealed. In the current study, we claimed that a mediating pattern could exist and the results supported our hypothesis. A significant possible mediating role of job-related negative affect was found in the relationship between workaholism and anxiety before sleep. This evidence confirmed that job-related negative affect experienced by workaholics might play a strategic role in determining the outcomes of workaholism, such as anxiety before sleep.

Regarding the interplay between workaholism and work engagement, recent theoretical conceptualization of HWI suggested that engaged workaholics might be protected from experiencing high health impairment derived from working compulsively and excessively, through the positive component of work engagement [11]. Although this interplay may help the comprehension of the HWI phenomena, apart from a previous empirical contribution by Van Beek and colleagues [6], research focused on this interplay seems to be neglected. In the current study, not only did we address this issue by examining a model where the interplay workaholism/work engagement may have a different impact on job-related negative affect, but we also postulated a focused hypothesis on the moderating role of gender on the relationship between the interplay workaholism/work engagement and job-related negative effect. In particular, we hypothesized that engaged workaholic women might be protected from experiencing job-related negative affect compared to disengaged workaholic women, and that this pattern would not be significant for men. Evidence supported our expectations and confirmed the buffering role that being engaged might play for workaholic women. As expected, since in our society being engaged at work is more difficult and challenging for a woman than for a man [80,101], coupled with the fact that women show greater emotion expression than men, and that men express more negative emotions than women [75,76,77], we can conclude that in our study the rewarding value of being engaged for women was greater than for men and protected them from experiencing higher job-related negative affect. Thus, our results supported the theoretical conceptualization of HWI provided by Loscalzo and Giannini [11], who call for more empirical contributions on the cross-conceptual binomial workaholism/work engagement. However, by adding a gender perspective to the Loscalzo and Giannini conceptualization, we included gender as a moderating variable and the results supported our expectations.

### 4.1. Limitations and Directions for Future Research

Although the current study provided interesting and original contributions to the HWI literature, several limitations should be taken into account. First, the cross-sectional nature of the study does not allow causal inference among the variables. Thus, although the mediating model at the core of the study stems from sound theoretical basis, the results should be interpreted with caution. However, the evidence found will provide useful insights for supporting the possibility of further studies to confirm the mediating model longitudinally. Additionally, since job-related affect consists in an ephemeral perception, which could be better depicted on a daily basis, diary-studies would probably be more adequate for future researches.

Second, since self-report measures were adopted, the results might be influenced by the participants’ acquiescence and need for social desirability and the emerged parameter estimates may have been contaminated by common method bias [102]. Despite Harman’s single-factor technique, adopting a confirmatory factorial analysis (CFA), was used to identify the risk of common method variance, a latent variable approach should be used in future studies, as recommended by William and McGonagle [103]. Future studies should also adopt multisource and objective measures. For example, as far as sleep disorders are concerned, actigraphy could be used for detecting more objective measures of quality and quantity of sleep. Regarding workaholism, data such as an observer-reported measure obtained by the partner or a colleague of the participants could be useful [104]. However, the problem is that it may be difficult for an observer to assess the feelings associated with working compulsively (e.g., feeling guilty when taking time off work), that is, the psychological and crucial component of workaholism.

Third, the snowball technique for data collection contravenes many of the assumptions supporting conventional notions of random selection and representativeness. However, it is not uncommon to use such a sampling strategy in organizational research [105].

Fourth, although we controlled the tested model for workload, job sector, tenure and gender, other individual variables highly correlated to workaholism, such as neuroticism and perfectionism, should have been included to better weight the impact of workaholism in the model.

### 4.2. Practical Implications

First, as Loscalzo and Giannini [11] claimed, making a distinction between engaged and disengaged workaholics is very important since it can help to prevent overpathologizing the act of working itself, which is a common and often socially valued behavior. This issue has also been recently raised by Billieux and colleagues [57], who criticized the current tendency to label virtually all human activities in terms of behavioral addictions and, therefore, as clinical pathologies. Since work is an important daily activity, and given that the stigma in organizations that could be associated with a diagnosis of workaholism might be associated with negative effects, we agree with Loscalzo and Giannini [11] in believing that it is important to make a clinical diagnosis of workaholism only when overworking is associated with low work engagement and high impairment, as usually required by the DSM criteria [106].

Second, according to Banihani and colleagues [80,101], it is important to recognize the fact that work engagement, which could play an important protective role in workaholic women, seems to be gendered and, therefore, managers should take into consideration that the myth of the “ideal worker” might be not suitable both for men and women. For instance, organizations should contrast the habit of implementing processes, practices, and interactions designed so that it is easier for men to experience psychological meaningfulness. Moreover, since men and women’s experiences in the workplace and at home may influence women’s capacity to be fully available and engaged in work, and men’s capacity to be available and engaged at home, tailored interventions should be undertaken to guarantee women’s availability at work, as well as their workplace safety perception. Since work engagement represents an important resource both for the organization’s productivity and for women’s health, particular attention should be paid to assure suitable organizational conditions to foster this construct.

## 5. Conclusions

In the current study, we provided evidence of a possible mechanism through which workaholism could impact on one of its outcomes, such as anxiety before sleep. Our results supported the possibility of a mediating role likely played by job-related negative affect in the relationship between workaholism and anxiety before sleep. Furthermore, we postulated a focused hypothesis on the moderating role of gender on the relationship between the interplay workaholism/work engagement and job-related negative effect. In particular, we hypothesized that engaged workaholic women might be protected from experiencing job-related negative affect compared to disengaged workaholic women, and that this pattern would not be significant for men. Evidence supported our expectations and confirmed the possible buffering role that being engaged may play for workaholic women.

## Figures and Tables

**Table 1:** Descriptives, inter-correlations and reliabilities of the study variables.

Variables	Mean	St. Dev.	1	2	3	4	5	6	7	8	9
1. Gender ^1,#^	-	-	-								
2. Age	45.65	11.61	−0.07	-							
3. Sector ^##^	-	-	−0.16	−0.40 ^2,^**	-						
4. Tenure	18.83	11.76	−0.10	0.80 **	−0.26 **	-					
5. Workaholism	2.46	0.51	−0.15	−0.22 **	0.34 **	−0.06	0.92				
6. Work engagement	4.95	0.99	0.15	−0.17 ^1,^*	−0.05	−0.01	0.12	0.93			
7. Job-related negative affect	2.35	0.87	−0.07	0.65	0.08	0.02	0.30 **	−0.20 *	0.89		
8. Anxiety before sleep	2.61	0.89	−0.02	0.21 *	0.10	0.21 *	0.26 **	−0.12	0.28 **	0.89	
9. Workload	3.73	0.75	−0.01	−0.11	0.19 *	−0.05	0.38 **	0.26 **	0.16	0.15	0.84

^1,#^ = Gender was coded as 1 = men and 2 = women; ^##^ = sector was coded as 1 = tertiary, 2 = secondary, and 3 = primary; ^2^ ** = *p* value < 0.001; ^3^ * = *p* value < 0.05.

**Table 2:** Anova one-way of the study variables on gender.

Variables	Gender	N	Media	F
Workaholism	Men	73	2.53	
	Women	73	2.38	3.542 ^§^
Work engagement	Men	73	4.79	
	Women	73	5.10	3.562 ^§^
Anxiety before sleep	Men	73	2.63	
	Women	73	2.59	0.06
Job-related negative affect	Men	73	3.51	
	Women	73	3.39	0.82
Workload	Men	73	3.74	
	Women	73	3.72	0.03

^§^ = *p* value 0.06.

**Table 3:** Results of the conditional process analysis.

Models	B	LLCI	ULCI	R^2^
Model 1: Mediation of job-related negative affect in the relationship between workaholism and anxiety before sleep Outcome variable: job-related negative affect				0.12 **
workaholism	0.53 **	0.21	0.84	
Covariate: workload	0.05	−0.75	0.87	
Covariate: age	0.02	0.09	−0.003	
Covariate: sector	0.06	−0.26	0.38	
Covariate: tenure	−0.01	−0.03	0.01	
Covariate: gender	−0.08	−0.37	0.21	
Model 1 bis: Mediation of job-related negative affect in the relationship between workaholism and anxiety before sleep Outcome variable: anxiety before sleep				0.19 **
Workaholism	0.36 *	0.03	0.69	
Job-related negative affect	0.19 *	0.02	0.37	
Covariate: workload	0.03	−0.17	0.23	
Covariate: age	0.02	−0.003	0.05	
Covariate: sector	25	−0.07	0.57	
Covariate: tenure	0.00	−0.02	0.02	
Covariate: gender	0.17	−0.12	0.46	
Indirect effect	0.10	0.01	0.24	
c. Model 2: Mediation model including interaction between workaholism and work engagement on job-related negative affect, moderated by gender Outcome variable: Job-related negative affect				0.25 **
Workaholism	0.69 **	0.37	10.01	
Work engagement	−0.30 **	−0.45	−0.14	
Gender	−0.02 **	−0.30	−0.26	
Workaholism *work engagement	−0.38 **	−0.67	−0.09	
Workaholism *gender	0.06	−0.52	0.64	
Work engagement *gender	0.04	−0.24	0.34	
Workaholism *work engagement *gender	−0.80 **	−1.40	−0.22	
Covariate: workload	0.17	−0.03	0.38	
Covariate: age	−0.00	−0.02	0.02	
Covariate: sector	−0.12	−0.43	0.20	
Covariate: Tenure	0.00	−0.01	0.02	
Test of conditional workaholism *work engagement interaction at value(s) of gender	Effect	F	p	
Men	0.02	0.02	0.88	
Women	−0.78	8.97	0.003	
d. Modello 2 bis: Interaction between workaholism and work engagement on job-related negative affect, moderated by gender Outcome variable: Anxiety before sleep				0.18 **
Workaholism	0.33 *	0.001	0.66	
Job-related negative affect	0.19 *	0.02	0.36	
Workload	0.04	−0.16	0.24	
Age	0.02	0.001	0.04	
Sector	0.21	−0.10	0.53	
Tenure Index of moderated mediation Index od conditional moderated mediation by work engagement Men	0.00 Index −0.15 0.003	−0.02 −0.39 −0.09	0.02 −0.006 0.07	
Women	0.50	−0.39	−0.02	

* = *p* < 0.05, ** = *p* < 0.001.
